# In-vivo handheld optoacoustic tomography of the human thyroid

**DOI:** 10.1016/j.pacs.2016.05.003

**Published:** 2016-06-27

**Authors:** Alexander Dima, Vasilis Ntziachristos

**Affiliations:** a Institute for Biological and Medical Imaging, Helmholtz Zentrum München, German Research Center for Environment and Health, Ingolstädter Landstrasse 1, Neuherberg 85764, Germany; b Chair for Biological Imaging, Technische Universität München, Arcisstrasse. 21, Munich 80333, Germany

## Abstract

We interrogated the application and imaging features obtained by non-invasive and handheld optoacoustic imaging of the thyroid in-vivo. Optoacoustics can offer complementary contrast to ultrasound, by resolving optical absorption-based and offering speckle-free imaging. In particular we inquired whether vascular structures could be better resolved using optoacoustics. For this reason we developed a compact handheld version of real-time multispectral optoacoustic tomography (MSOT) using a detector adapted to the dimensions and overall geometry of the human neck. For delivering high-fidelity performance, a curved ultrasound array was employed. The feasibility of handheld thyroid MSOT was assessed on healthy human volunteers at single wavelength. The results were contrasted to ultrasound and Doppler ultrasound images obtained from the same volunteers. Imaging findings demonstrate the overall MSOT utility to accurately retrieve optical features consistent with the thyroid anatomy and the morphology of surrounding structures.

## 1 Introduction

Multispectral optoacoustic tomography (MSOT) can visualize functional and molecular parameters of tissue at high resolution while also reaching tissue depths of several mm to few cm [1]. Using targeted contrast agents the modality enables high sensitivity and specificity to molecular parameters of disease. Several studies have highlighted possible applications of the technique in a clinical context, including follicular thyroid carcinoma [2], cardio vascular disease [3], [4] and arthritis [5]. MSOT thereby operates on optoacoustic images acquired at different optical excitation wavelengths to identify contrast agents that otherwise fail to generate sufficient signal strength to be discernible from the intrinsic background absorption (e.g. hemoglobin). In turn, optoacoustic images are produced by shining light of transient intensity onto tissue and detecting ultrasound signals generated by thermoelastic expansion of structures that have absorbed the incident light energy. By operating in the near-infrared, it was shown that vascular structures can be imaged up to 4 cm deep inside tissue. In particular, images of the human carotids [6] and other deep-seated vascular structures [7], [8], [9] have been shown.

Handheld implementation of MSOT is of particular interest in a clinical setting as it allows flexible utilization at the point of care. For this reason previous designs of handheld optoacoustic scanners attempted the extension of common linear ultrasound arrays by optical components (laser and light guides) to also enable combined optoacoustic and sonography imaging. In 2005 the first such system was introduced [10] with subsequent designs attempting various improvements to achieve better frame rates, penetration depth or signal-to-noise ratio [11], [12], [13], [14]. Advantages of this design pattern include good availability of components such as detection arrays and acquisition electronics and simple co-registration with the sonographic image. On the other hand, the design suffers from low image quality due to strong artifacts and low transversal resolution (i.e. parallel to the array axis), which also limit the achievable imaging depth in tissue.

We have shown in [6] that the image performance in hand-held operation improves significantly when using curved ultrasound arrays, as opposed to linear arrays more commonly used in ultrasound imaging. Sonography employs beam-forming techniques to shape the excitation pulse and focus on the region of interest when detecting highly directive echo signals. In contrast, optoacoustic imaging has only limited capacity to actively determine the tissue volume to be excited, because photon scattering rapidly renders any light focused at the tissue surface diffusive. Furthermore, optoacoustic waves generally (i.e. in case of point sources) propagate in all directions and ideally require detection on a spherical surface completely enclosing the illuminated volume. Hence, the image quality achieved depends largely on the geometry of the detection array. Curved arrays allow the collection of a more complete projection data-set which can be employed together with model-based inversion algorithms [15] to more accurately represent the underlying light-absorption distribution.

Optoacoustic imaging, considered in handheld mode, can visualize a new set of human tissue features by resolving absorbing structures with resolution similar to the one achieved by clinical ultrasound imaging. In this work we interrogated features that could be visualized when imaging the thyroid. In particular we were interested in investigating anatomical markers associated with optoacoustic thyroid imaging and comparing the relative performance of optoacoustic and ultrasound imaging when focusing on anatomical and vascular structures.

## 2 Materials and methods

**Fig. 1:**
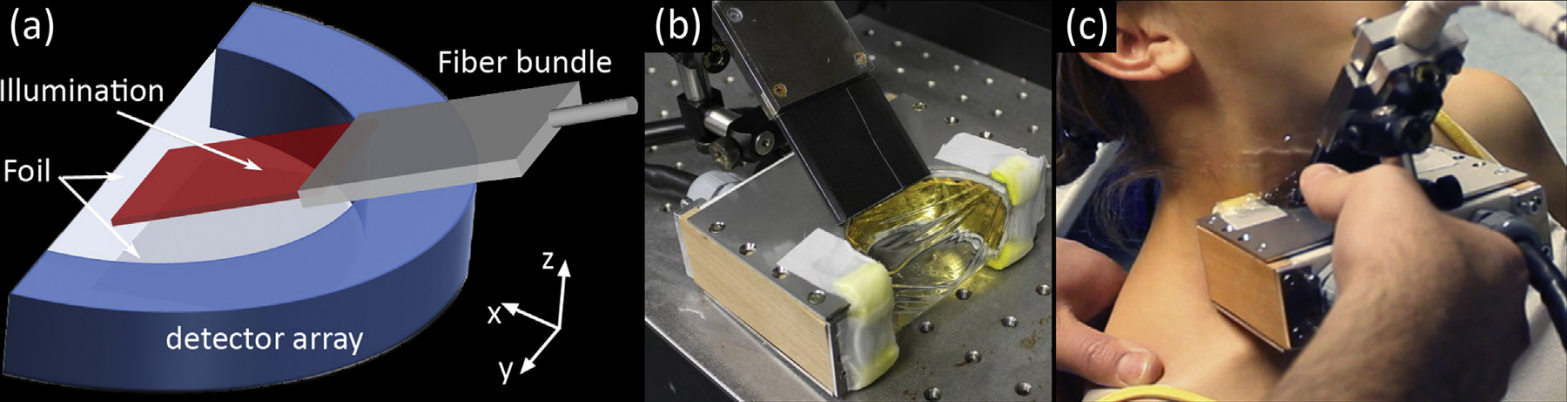
Non-invasive handheld optoacoustic probe: (a) schematic, (b) implementation, (c) operational use.

The optoacoustic system employed in this study was based on a previously described multispectral optoacoustic tomographic device [6] modified by improved illumination (laser and fiber bundle) and mechanical components for optimal water coupling and handheld operation. In particular, the previously chosen fiber bundle and optical diffuser combination was replaced by a custom tailored solution that provided a sharper illumination stripe on the skin surface. Fig. 1 shows the schematic of the handheld measurement probe, its implementation and operational use. The curved detector array consisted of 64 individual elements with a common radius of 40 mm and angular span of 172° within the x-y plane. Elements were mechanically focused in elevation (z-axis) and capable of detecting optoacoustic signals at frequencies of up to 7.5 MHz. The array was connected to a 12-bit custom made data acquisition (DAQ) system digitizing all channels in parallel at 40 MSamples/s. An in depth characterization of the array can be found in [15]. For optimal acoustic coupling the array cavity was filled with de-ionized water and sealed using acoustically and optically transparent foil. This highly flexible foil in combination with ultrasound gel, to lubricate contact between foil and human skin, enabled excellent coupling to the human body. A double O-ring sealing gasket ensured leak-proof operation. Optical excitation was provided by a (slow) tunable pulsed laser system, SpitLight 600 OPO (InnoLas Laser GmbH, Germany), which enabled a repetition rate of 10 Hz. Light delivery was facilitated by a custom made fiber bundle with rectangular output of 40 × 0.9 mm^2^ size (CeramOptec GmbH, Germany). The fiber bundle was mounted on the detection array such that an adjustable angle was formed to the x–y plane. For optimal results the beam was always directed at the array middle in z and illuminated an elongated stripe of approximately 50 × 5 mm^2^ size. The maximum light fluence measured on the tissue at the wavelength employed (800 nm) never exceeded 20 mJ/cm^2^.

Image reconstruction from the digitized acoustic data was performed in two ways. During the measurement sessions live imaging was enabled using a delay and sum algorithm implemented on a high-performance graphics card (<30 ms/image). A reconstructed view of 40 × 40 mm^2^ at 600 × 600 pixels allowed optimal navigation and probe positioning during the measurement. To increase accuracy and image quality the same view was later reconstructed using a model-matrix inversion scheme [16], which had previously been shown to yield superior imaging results [17], [18]. In addition optoacoustic images were post-processed by window and leveling to improve contrast.

**Fig. 2:**
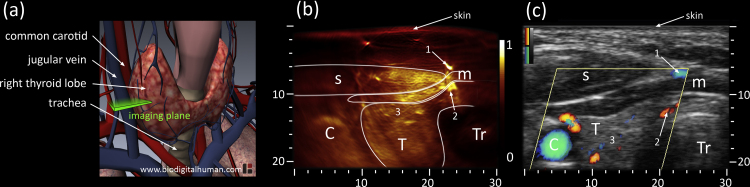
(a) Anatomy of thyroid gland including cardio-vascular and respiratory system; the cross-sectional 2D imaging plane is highlighted in green. (b) Optoacoustic and (c) ultrasound cross-sections of the left thyroid lobe of the first volunteer. The optoacoustic image, leveled and normalized from 0 to 1, shows with high sensitivity vascular features of skin, muscles [1], [2] and within the thyroid lobe [3]. The corresponding ultrasound image in grayscale also depicts Directional Power Doppler signals superimposed in color (red/blue colormaps indicating opposite flow directions). C: Carotid, T: Thyroid, Tr: Trachea, s: sternocleidomastoid muscle, m: infrahyoid muscle; axes in mm.

In this study we focused on the thyroid lobes in healthy human volunteers. Fig. 2(a)shows a schematic anatomical depiction of a human thyroid in situ. We attempted cross-sectional imaging at the location marked “imaging plane”. Two healthy female volunteers were imaged using the same detector and configuration, each scan following the same protocol. The measurement protocol was split in two imaging sessions, optoacoustic and ultrasound imaging. First an optoacoustic scan involved imaging of both lobes and the connecting isthmus to understand thyroid anatomy, depicted in Fig. 1(c). In a second session the same procedure was repeated using a commercial ultrasound system, Terason 2000+ (Teratech Corp., USA). The ultrasound scan revealed thyroid anatomy and included Directional Power Doppler to better visualize vasculature based on directional flow measurement.

## 3 Results

For each volunteer a large optoacoustic dataset was produced (>2000 images) and validated by Directional Power Doppler and echo-ultrasound, recording short image sequences at corresponding positions. For each ultrasound position a matching set of optoacoustic images could be identified by manual inspection. To facilitate a concise description of results, we show here the same position as indicated in Fig. 2(a) for both thyroid lobes, each from a different volunteer.

Fig. 2 further shows results from the left thyroid lobe of the first volunteer. The optoacoustic image, depicted in Fig. 2(b), allows immediate identification of large features characteristic of the anterior neck such as the carotid artery and the trachea, which appears round and dark as ultrasound cannot penetrate or escape from air filled cavities. Similarly, high vascularization within muscles allows identification of the sternocleidomastoid and an infrahyoid muscle. Although deeper seated the thyroid lobe can also be distinguished. In particular thyroid vascularization within the anterior part is clearly visible as indicated by the group of vessels surrounding marker 3. Vessels appearing as round bright dots traverse the imaging plain in a perpendicular angle while others, elongated or dashed in shape, cross through the imaging plane at lower angles. To validate these findings Fig. 2(c) depicts the corresponding echo-ultrasound image in grayscale with Directional Power Doppler signals superimposed in color (red/blue colormaps representing opposite flow directions). Anatomical landmarks, such as trachea, carotid artery and muscles, confirm the optoacoustic assessment. Size and position of the thyroid lobe also coincide with the optoacoustic result. Vascularization however does not provide sufficient echo and thus requires application of Doppler ultrasound to identify vessels by measuring blood flow. In Fig. 2(c) the Doppler signal is measured within the yellow rhombus and confirms two major findings from Fig. 2(b): two strong vessels above (marked 1) and below (marked 2) the infrahyoid muscle as well as various vessels within the anterior part of the thyroid (surrounding marker 3) of different size and flow directions.

**Fig. 3:**
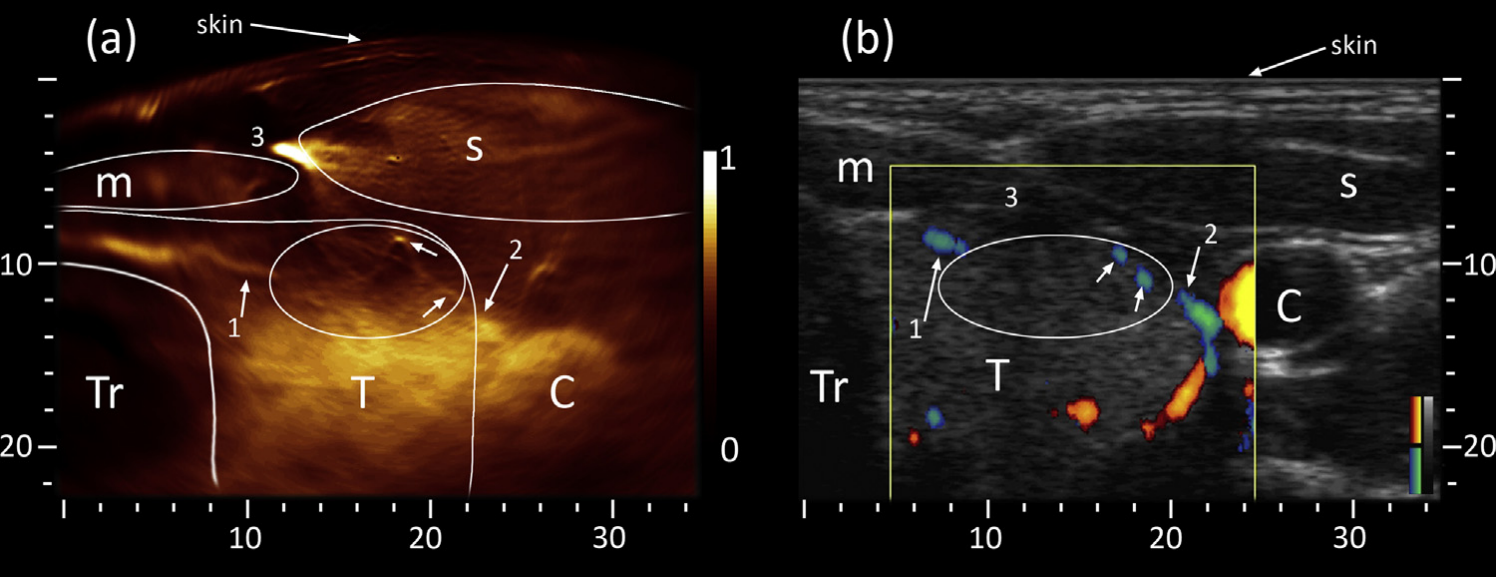
Cross-section of right thyroid lobe of the second volunteer. (a) To limit the influence of strong absorbers (large vein marked 3) on available contrast the optoacoustic image was range limited with respect to high intensity values before leveling and normalization from 0 to 1. Besides larger features (e.g. muscles, thyroid) optoacoustic imaging allows identification of prominent vascularization [1], [2] and a fine vascular network (oval highlight) stretching inside the imaging plane and perpendicular to it (fine dots). (b) The corresponding ultrasound image confirms features detected in (a), whereby grayscale represents the pulse-echo and color the Directional Power Doppler signal (red/blue colormaps indicating opposite flow directions). C: Carotid, T: Thyroid, Tr: Trachea, s: sternocleidomastoid muscle, m: infrahyoid muscle; axes in mm.

Results from the right thyroid lobe of the second volunteer are shown in Fig. 3. During the optoacoustic scan, depicted in Fig. 3(a), the sternocleidomastoid muscle was tense and therefore pushed the carotid artery and the thyroid lobe further from the curved skin surface. An infrahyoid muscle is also discernible, however due to its peripheral position at reduced resolution and intensity. The thyroid lobe, taking up the space between trachea and carotid artery, is clearly visible. Three prominent vascular features have been highlighted as they extend mostly inside the imaging plane. An elongated vessel (marked 1) stretches from the isthmus towards the anterior part of the thyroid lobe. A second vessel (marked 2) similarly reaches from the vicinity of the carotid artery towards the same area. To highlight patterns within this anterior part we have encircled the area with an ellipse and pointed out two features. The vessel marked as “2” appears to continue within the ellipse (lower arrow) extending towards the bright spot (upper arrow) while also branching. Similar patterns can be found when following the left vessel (marked 1), however at lower signal strength and contrast. In turn ultrasound validation using Fig. 3(b) is straight forward for large features such as the muscles, trachea or carotid artery. Large vasculature previously identified in the optoacoustic image (marked 1 and 2) can also be found using Directional Power Doppler, however at lower resolution and sensitivity due to the indirect measurement based on blood flow. Detecting weak flow in the presence of movement (of probe and patient) is generally difficult and highly operator dependent, which explains the better resolution and more coherent vascular appearance in Fig. 3(a). The same argument can be made for the large vein (marked 3) that generated the strongest optoacoustic signal, but is missing from Fig. 3(b). We suspect that a combination of applied pressure and chosen Doppler parameters resulted in blood flow too weak to detect. Nonetheless, sufficient echogenicity of the thyroid allows approximate placement of the missing vein in between the two muscles and just above the thyroid lobe. The fine vascular network that we observed in the optoacoustic image (oval highlight) can only be inferred from the echo-ultrasound image as dark spots and patches, again providing too weak flow to yield a Doppler signal.

## 4 Discussion

Thyroid nodules have a high and rising incidence in the general population [19], [20] and imaging is employed as part of the clinical disease management. Neck ultrasonography is routinely applied for nodule detection. A suspicious ultrasound result may be further examined by radioactive 2-deoxy-2[^18^F]fluoro-d-glucose positron emission tomography (FDG-PET) or followed up by fine needle aspiration (FNA). Optical imaging techniques, such as optical coherence tomography or elastic light-scattering spectroscopy, have been suggested for differentiating benign from malignant thyroid nodules [21], [22]. However, limitations in terms of depth imposed by the strong optical scattering in tissue prevent their clinical non-invasive imaging application.

Herein, we improved a previously developed optoacoustic handheld imaging system for investigating optoacoustic imaging of the human thyroid. The resulting system offered a light-weight head to allow handheld operation using a single hand. Further engineering can nevertheless reduce the weight and overall form of the scan-head. Subsequently, we examined the anterior-side of the neck of healthy human volunteers in the thyroid area. In both volunteers we were able to detect the outline of the thyroid and also identify vascular features at tissue depths up to 20 mm below the skin. For subsequent validation we employed echo-ultrasound and Doppler ultrasound imaging. Large and medium sized vessels, detected using Doppler ultrasound, were captured by the optoacoustic system at better resolution. Additionally, the optoacoustic images revealed finer vascular networks undetected by Doppler ultrasound and provided a more complete picture of thyroid vascularization.

Reliable vascular information (over- or under-expression) can yield important clues in the diagnosis of thyroid disease [23], in particular when combined with other diagnostic modalities such as ultrasound. When deciding malignancy of thyroid nodules a high sensitivity to vascularization is especially desirable [24]. Furthermore, subsequent fine needle aspiration can also be performed using optoacoustic needle guidance [13]. On the other hand, multi-spectral acquisition could further increase accuracy by for example including tissue oxygenation as a diagnostic parameter. A study already demonstrated the capability of MSOT to differentiate between benign and malignant nodules in diseased thyroid samples after thyroidectomy [25].

Despite the potential of multispectral acquisition we chose to focus our investigation on the achievable imaging quality at single-wavelength − the essential input dataset to all unmixing algorithms. Indeed, for unmixing algorithms to reach a high level of acuity and sensitivity high-quality input data with respect to two features is required: 1) image quality (spatial resolution and artifacts); 2) motion stability (target and operator movement between multiple laser pulses). Due to significant movement involved in handheld imaging of patients at the point of care and insufficient repetition rate and tuning speed of the employed laser, we chose to study and optimize the MSOT system for image quality first. Subsequent developments will deal with the equally challenging task of minimizing motion influence and developing appropriate unmixing solutions.

To allow efficient navigation during screening and for needle guidance a large field of view is necessary. Here we have chosen a view comparable to that of ultrasound even though the employed detection array enabled high resolution imaging only within a much smaller area. In the employed configuration the skin was positioned approximately 25–30 mm from the central transducer element, which enabled the best resolution and sensitivity from 5 to 20 mm below the skin surface. This limitation is imposed by the array radius (too small) and the width of individual transducer elements (too large). Thus, further probe development will focus on extending the radius of array curvature while increasing the number of elements and shrinking their size, comparable to arrays studied in [15]. This should further suppress imaging artifacts and increase resolution throughout a large field of view. In terms of fully exploiting the potential of MSOT, we expect the application of improved laser sources [7] and molecularly targeted contrast agents [2] to allow detection of malignant lesions based on molecular features. Ultimately, successfully applying molecular imaging techniques in the diagnosis and treatment of thyroid disease will require further research towards quantitative evaluation.

## Conflict of interest statement

The authors declare that there are no conflicts of interest.
